# Radiological Cardiothoracic Ratio as a Potential Marker of Left Ventricular Hypertrophy Assessed by Echocardiography

**DOI:** 10.1155/2022/4931945

**Published:** 2022-06-15

**Authors:** Krystian Truszkiewicz, Piotr Macek, Małgorzata Poręba, Rafał Poręba, Paweł Gać

**Affiliations:** ^1^Center for Diagnostic Imaging, University Clinical Hospital in Wroclaw, Wroclaw, Poland; ^2^Department of Internal Medicine, Occupational Diseases and Hypertension, Wroclaw Medical University, Wroclaw, Poland; ^3^Department of Paralympic Sports, Wroclaw University of Health and Sport Sciences, Wroclaw, Poland; ^4^Centre for Diagnostic Imaging, 4th Military Hospital, Wroclaw, Poland; ^5^Division of Environmental Health and Occupational Medicine, Department of Population Health, Wroclaw Medical University, Wroclaw, Poland

## Abstract

The aim of the study was to verify the usefulness of the radiological cardiothoracic ratio as a potential marker of left ventricular hypertrophy assessed by echocardiography. The study included 96 patients (mean age: 49.52 ± 9.64 years). Chest radiograph in the PA projection and echocardiography were performed. In CR the measurement of the cardiothoracic ratio (CTR) was performed. Assuming CTR > 0.50, heart silhouette enlargement was diagnosed. In echocardiography, four types of left ventricular geometry were assessed: normal geometry (NG), concentric remodeling (CR), concentric hypertrophy (CH), and eccentric hypertrophy (EH). It was shown that patients with an enlarged heart silhouette were characterized by a significantly more frequent occurrence of left ventricular hypertrophy (LVH) on echocardiography than patients with a nonenlarged heart silhouette. In the subgroup of patients with LVH compared to the subgroup of patients with normal left ventricular geometry, CTR values are statistically significantly higher, and heart silhouette enlargement is significantly more frequent. The criterion “CTR > 0.49” estimates LVH with a sensitivity of 93.3% and specificity of 82.7%, which translates into a high accuracy of 84.4%. By analyzing the prediction of left ventricular geometry types, high accuracy of CH prediction was obtained using the “CTR > 0.49” criterion of 80.2% (with a high sensitivity of 84.0% and a satisfactory specificity of 60.0%) and a high accuracy of EH prediction using the “CTR > 0.52” criterion of 71.9% (with high sensitivity 80.5% and low specificity 36.8%), as well as low CR prediction accuracy of only 57.3% (with low sensitivity 36.7%, even if high specificity 78.7%). In summary, the radiological cardiothoracic ratio may be a moderate marker of left ventricular hypertrophy assessed according to standard echocardiographic criteria, provided that its cut-off point is standardized in each population of subjects.

## 1. Introduction

The cardiothoracic ratio (CTR) is defined as the ratio of the greatest transverse dimension of the heart (C weight) to the greatest dimension of the chest measured to the inner edge of the ribs (T weight) on the chest PA radiograph [1]. Normal CTR values were ≤0.5; with values >0.5, enlargement of the heart silhouette is suggested. Due to the high variability in the exposure, the patient's position, and the distance of the X-ray tube, for routine CTR measurements, no other than PA chest projection should be used, and if necessary, their limitations should be kept in mind [2–5].

CTR is a widely used, time-saving, and non-cost-effective indicator when interpreting a chest X-ray to assess the size of the heart shape. In the literature, we find publications both about its important value and discrediting its importance in common use.

A large, prospective cohort study on a group of almost 3.5 thousand 4-year follow-ups showed that in people undergoing hemodialysis, higher CTR is associated with a higher risk for both general and cardiovascular reasons [6]. In the group of hemodialysis patients, it has also been shown that CTR > 55% is an important independent factor influencing the 2-year all-cause mortality [7]. Another study conducted on a group of almost 1.8 thousand 4-year follow-ups has shown that CTR as a simple indicator is a strong predictor of poor prognosis in patients with rheumatic heart disease undergoing heart valve replacement [8]. CTR in patients with chronic heart failure can be used to stratify cardiovascular risk, and its value >0.5 is associated with higher mortality and an increase in hospitalizations [9].

Despite the potential clinical applications of CTR described above, a common opinion is that CTR is of low clinical usefulness, which is also reflected in some studies that emphasize, for example, a weak correlation between CTR and the parameters of heart function [10–12]. Another study in a group of patients undergoing coronary angiography questioned the validity of the normal cut-off point of 0.5, showing increased mortality in patients undergoing coronary angiography at 0.42 < CTR < 0.49 [13]. A previous study showed that despite the easy determination of the parameter and high repeatability between researchers/clinicians, it has low accuracy as a method of distinguishing the normal size of the heart from an enlarged one based on a single cut-off point [14]. In the literature, however, we find little data on the real clinical value of CTR, and there are no studies that would unambiguously tip the balance on one side.

When talking about the “large” left ventricular (LV), we should distinguish at least two terms describing it, namely, hypertrophy and dilatation. LV hypertrophy is an increase in the mass of the left ventricular secondary to an increase in the thickness of the left ventricular walls. The overgrown left ventricular, in addition to increasing the mass of the miokardium, may be of the correct size or enlarged [15, 16]. Left ventricular dilatation is understood as an increase in the internal dimensions of the left ventricular cavity above normal [17].

The main causes of left ventricular hypertrophy (LVH) are arterial hypertension, significant narrowing of the renal arteries, the so-called athlete's heart, aortic stenosis, aortic coarctation, hypertrophic cardiomyopathy, and aortic/mitral valve regurgitation. All the above-mentioned diseases, in connection with the increase in left ventricular volume load in certain stages, may lead to left ventricular dilatation [16, 18]. Echocardiography is the most accessible and routinely used method in clinical management to estimate the size of the left ventricular.

The aim of the study was to verify the usefulness of the radiological cardiothoracic ratio as a potential marker of left ventricular hypertrophy assessed by echocardiography.

## 2. Materials and Methods

The study included 96 patients examined in the cardiology clinic recruited for the study based on the inclusion and exclusion criteria. The inclusion criteria for the study were as follows: age ≥ 18 years, clinical indications for echocardiography and chest radiograph in the PA projection, and informed written consent to participate in the study. The exclusion criteria from the study were a large volume of fluid in the pleural cavity/cavities, the presence of fluid in the pericardial sac, the coexistence of chronic respiratory diseases (COPD, bronchial asthma, and interstitial lung diseases), past pneumonia in the past 6 months, cancer history, coexistence of diseases systemic diseases, history of congenital heart defects, previous thoracic, cardiosurgical and neurosurgical procedures in the thoracic spine, and changes in the therapy of chronic diseases in the past 6 months. The required sample size of 96 people was determined using a sample size calculator, adopting the following calculation criteria: population size 2 million, fraction size 0.5, maximum error 10%, and confidence level 95%. The mean age in the study group was 49.52 ± 9.64 years, height 1.67 ± 0.09 m, body mass 73.12 ± 9.88 kg, and BMI 26.30 ± 2.78 kg/m^2^. The general characteristics of the entire study group are presented in Table 1.

The study was conducted as part of a research project entitled “Radiological cardiothoracic ratio as a predictor of heart size as assessed by echocardiography, computed tomography and magnetic resonance imaging.” The assumptions and protocol of the study were positively assessed by the local institutional bioethics committee (KB–414/2021).

Basic anthropometric measurements were measured in all patients, and imaging examinations were performed: chest radiograph in the PA projection and echocardiography.

The chest radiograph was performed using the standard method in the standing position in the posterior-anterior (PA) view. The radiograph was taken with a breath hold during the maximal inspiration phase, using a kilovolt of the 120 kV X-ray tube. All radiographs met the radiological criteria for the correct acquisition of the X-ray image.

**Figure 1:**
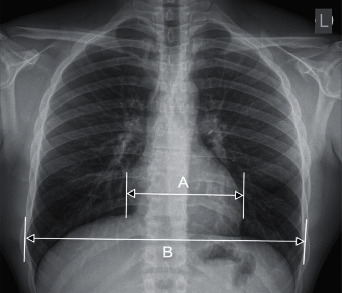
The method of measuring CTR on a chest radiograph in the PA projection. CTR = A/B.

For the purposes of the current study of retrospective CTR measurements in all chest radiographs performed, one specialist in radiology and imaging diagnostics, holding institutional individual certification in the field of cardiovascular radiology, performed it. The measurement of the cardiothoracic ratio (CTR) was performed using a diagnostic radiological station that met the applicable legal requirements for the assessment of X-rays. The maximum width of the heart's silhouette (C width) and the maximum width of the chest (T width) were measured. The CTR value was obtained by dividing the measured C width value by the T width value. Assuming CTR > 0.50, enlargement of the heart silhouette was diagnosed. The method of measuring CTR on a chest radiograph in the PA projection is presented in Figure 1.

Transthoracic echocardiography was performed using a standard examination protocol using the ALOKA ProSound 6 (Aloka Inc., Tokyo, Japan). Using the M-mode presentation under the control of a two-dimensional examination (in the parasternal projection in the long axis of the left ventricular), the dimensions of the lumen and the thickness of the walls of the left ventricular were measured: left ventricular end-diastolic diameter (LVEDd), left ventricular end-systolic diameter (LVESd), interventricular septum diastolic diameter (IVSDd), and posterior wall diastolic diameter (PWDd). Left ventricular dimensions and left ventricular wall thickness were used to estimate left ventricular mass (LVM), left ventricular mass index (LVMI), and relative wall thickness (RWT). The following mathematical formulas were used in the estimation: LVM = 0.8 × [1.04 × (LVEDd + PWDd + IVSDd)^3^ -LVEDd^3^] + 0.6; LVMI = LVM/BSA (where BSA was the body surface area calculated from Du Bois' formula: BSA = 0.007184 × body weight ^0.425^ × height^0.725^); RWT = (IVSDd + PWDd)/LVEDd.

The estimated LVMI and RWT values were used as criteria for classifying four types of left ventricular geometry: normal geometry (NG), concentric remodeling (CR), concentric hypertrophy (CH), and eccentric hypertrophy (EH). NG was diagnosed when RWT ≤ 0.45 and LVMI ≤ 125 g/m^2^ in men or ≤ 110 g/m^2^ in women; CR when RWT > 0.45 and LVMI ≤ 125 g/m^2^ in men or ≤ 110 g/m^2^ in women; CH when RWT > 0.45 and LVMI > 125 g/m^2^ in men or > 110 g/m^2^ in women; EH when RWT ≤ 0.45; LVMI > 125 g/m^2^ in men or > 110 g/m^2^ in women. Moreover, patients diagnosed with CR, CH, and EH were classified into the general group of patients with left ventricular hypertrophy (LVH). For the purposes of the present study, all echocardiographic examinations were performed by one specialist in cardiology with 20 years of experience.

Echocardiographic examination and chest radiograph were performed in the shortest possible time interval, not longer than 7 days. The mean time interval between the performed imaging examinations was 4.32 ± 1.03 days.

Statistical analysis was performed using the Dell Statistica 13 statistical package (Dell Inc., USA). For quantitative variables, arithmetic means and standard deviations were calculated. The W Shapiro–Wilk test was used to check the normality of the distribution of variables. The null hypotheses for normally distributed quantitative independent variables were tested with the *t*-test. The null hypotheses for nonnormally distributed variables were tested with the Mann–Whitney *U* test. For qualitative variables, percentages were calculated. The null hypotheses for qualitative variables were tested with the chi-square test. To determine the potential linear relationships between the analyzed quantitative variables, a correlation analysis was performed. Pearson's correlation coefficients were established for quantitative variables with a normally distributed, and Spearman's correlation coefficients for quantitative variables with a distribution other than normal. Moreover, an analysis of the prediction accuracy assessment was performed, in which the proposed cut-off points for the predictors were estimated based on ROC (Receiver Operating Characteristic) curves. The level of statistical significance was *p* < 0.05.

## 3. Results

LVMI in the studied group of patients was 107.80 ± 31.05 g/m^2^, while the RWT was -0.48 ± 0.07. Left ventricular hypertrophy (LVH) was found in 84.4% of patients, CR in 49.0% of patients, CH in 15.6%, and EH in 19.8% of patients. The study group was characterized by a CTR of 0.51 ± 0.04. Enlarged heart silhouette was diagnosed in 30.2% of patients. Selected variables from imaging studies are summarized in Table 2.

Comparing the subgroups of patients distinguished based on the CTR criterion, it was shown that patients with an enlarged heart silhouette on the chest radiograph in the PA projection were characterized by a significantly more frequent occurrence of left ventricular hypertrophy (LVH) on echocardiography than patients with a nonenlarged heart silhouette. There is a significant difference between patients with an enlarged heart silhouette and patients with a nonenlarged heart silhouette of the more frequent incidence of left ventricular concentric hypertrophy in the first of these subgroups. The left ventricular geometry in the subgroups distinguished based on the heart silhouette enlargement criterion is presented in Table 3.

In a comparative analysis of the subgroups distinguished based on the presence of LVH, it was found that in the subgroup of patients with LVH compared to the subgroup of patients with normal left ventricular geometry, CTR values are statistically significantly higher, and heart silhouette enlargement is significantly more frequent.

When comparing the subgroups of patients with different types of left ventricular geometry in echocardiography, statistically significant higher CTR values were observed in the CR, CH, and EH subgroups than in the NG subgroup and also in the CH and EH subgroups than in the CR subgroup. Moreover, heart silhouette enlargement was significantly more frequent in the subgroup of patients with CR, CH, and EH than in the subgroup of patients with NG and also in the subgroup of patients with CH than in the subgroup of patients with CR. The values of the cardiothoracic ratio in the subgroups distinguished based on the geometry of the left ventricular are presented in Table 4.

The correlation analysis showed the existence of statistically significant positive linear relationships between LVEDd and CTR (*r* = 0.38, *p* < 0.05), LVM and CTR (*r* = 0.42, *p* < 0.05), and LVMI and CTR (*r* = 0.50, *p* < 0.05), Table 5.

**Figure 2:**
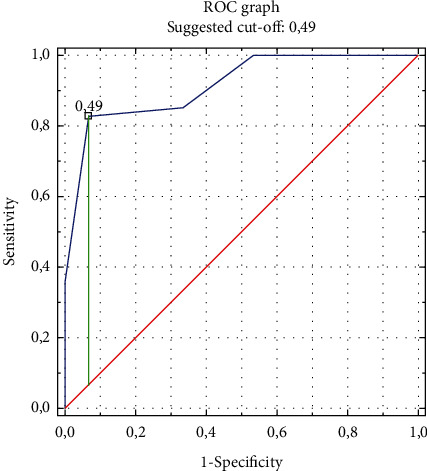
ROC curve for predicting left ventricular hypertrophy using the CTR value on the chest radiograph.

**Figure 3:**
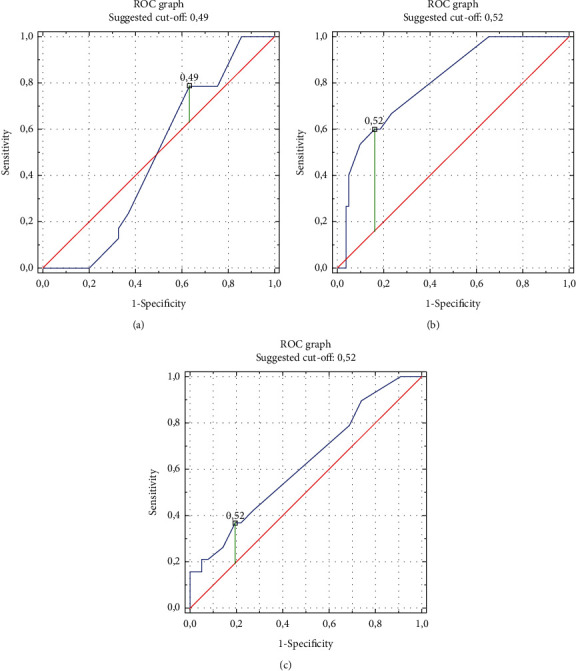
ROC curves for predicting left ventricular geometry using the CTR value on the chest radiograph. (a) CR: concentric remodeling of the left ventricular. (b) CH: concentric hypertrophy of the left ventricular. (c) EH: eccentric hypertrophy of the left ventricular.

The sensitivity and specificity analysis was used to assess the accuracy of CTR values as a predictor of left ventricular hypertrophy and types of left ventricular geometry. The results of the sensitivity and specificity analysis of the standard criterion “CTR > 0.50” (defining the heart silhouette enlargement on the chest radiograph in the PA projection) and the CTR criteria determined based on the performed ROC curves presented in Figures 2 and 3 are summarized in Table 6.

The CTR criteria established based on the ROC curves made were each time characterized by higher predictive accuracy than the standard CTR criterion defining the heart silhouette enlargement. In the studied group of patients, the criterion “CTR > 0.49” estimates LVH with a sensitivity of 93.3% and specificity of 82.7%, which translates into a high accuracy of 84.4%. By analyzing the prediction of left ventricular geometry types, high accuracy of CH prediction was obtained using the “CTR > 0.49” criterion of 80.2% (with a high sensitivity of 84.0% and a satisfactory specificity of 60.0%) and a high accuracy of EH prediction using the “CTR > 0.52” criterion of 71.9% (with high sensitivity 80.5% and low specificity 36.8%), as well as low CR prediction accuracy of only 57.3% (with low sensitivity 36.7%, even if high specificity 78.7%).

## 4. Discussion

The results of our research have shown that CTR may be a helpful indicator in the prediction of LVH, especially in the subgroup of patients with concentric hypertrophy. High sensitivity, specificity, and accuracy should be emphasized while maintaining the CTR > 0.49 condition. Based on the compiled data, it can be concluded that each time CTR > 0.49 should be associated with the consequence of further investment regarding, in particular, the thickness of the LV walls. However, there are few studies in the literature comparing echocardiographic and CTR values in the assessment of LV size and its wall thickness. In 2006, a publication was published that investigated the correlation of a chest radiograph and a transthoracic ultrasound of the heart in the assessment of cardiomegaly in patients with arterial hypertension. Researchers have demonstrated, like us, that there is a significant correlation (*p* < 0.05) between LV hypertrophy and CTR while indicating a weak correlation between CTR and LV enlargement [19]. Similarly, it was demonstrated in the study of cardiomegaly during Chagas disease (no correlation between the increased LVEDD size and cardiomegaly was determined in CTR) [20].

Other conclusions were presented in the article, which tried to answer the question of whether the cardiomegaly described by CTR is the same as the cardiomegaly described in echocardiography based on the observation of patients with NSTEMI. This study demonstrated a similar negative and positive prediction of cardiomegaly in CTR, indicating that all patients with suspected cardiac enlargement should have an echo. As a limitation of the study, one should consider the specifics of the study group and (which was also noticed by the researchers) assess whether this situation also applies to the wider population [21]. In the context of this information, the strength of our study should be considered to select a more diverse population.

Comparing our research group with the group studied by Costa et al. [22], in completely different populations (patients of cardiology clinics vs. patients with chronic kidney disease with hemodialysis introduced for at least six months), a similar percentage of patients with LVH was obtained (84.4% vs. 83.0%); however, the analysis of data on the distribution of certain types of geometry indicates the existing differences in the studied groups. Both were dominated by concentric overgrowth (the sum of CR and CH in our population is, i.e., 64.6% vs. 67.4% in the cited study), but the distribution of eccentric hypertrophy was slightly different (19.8% vs. 32.6%). Both studies proved the usefulness of diagnostic CTR in determining LVH. Similar conclusions were presented in the study that investigated the prevalence of hypertension, calcification in the heart valves, and LVH in patients undergoing peritoneal dialysis. Researchers have shown that a separate subgroup of patients with LVH has a significantly higher CTR and that LVMI significantly correlates with CTR [23].

A study that assessed the clinical manifestations and course of HCM in the pediatric population showed an increased CTR (all study participants had an average CTR > 0.65), and echocardiography confirmed different LVH morphologies in all patients [24], which may also indirectly prove the usefulness of CTR in determining LVH in the pediatric population. In our opinion, however, it is necessary to demonstrate great caution in determining and interpreting CTR in the pediatric population, especially in the context of the findings of the work, where no correlation has been demonstrated between CTR and LV measurements in echocardiography and the severity of LVH in children with end-stage renal disease and anemia [25].

Researchers from Korea tried to create a system based on several criteria (including electrocardiography and CTR) that would allow effective prediction of LVH among asymptomatic hypertensive patients. The researchers emphasized the high negative predictive value of CTR in detecting LVH, and the CTR introduced into their method as an additional risk factor significantly improved the accuracy of the LVH suspicion [26]. Similar conclusions were reached by researchers from Brazil who proved that the assessment of the heart shape on radiographs (AP and lateral) and the assessment of the electrocardiogram show great value in the prognosis of LVH in patients with arterial hypertension. Moreover, they developed the thesis that such a set of tests should be routinely performed in this group of patients to monitor the occurrence of possible LVH [27]. It is important to remember that in the subsequent stages of LV remodeling during arterial hypertension and other diseases that, the final stage may lead to its significant enlargement (LVD). A review and pooled analysis of CTR in LVD prediction has shown that increased CTR has no value in LVD prediction [28].

This is another finding of our research on the predictive suitability of CTR. We previously demonstrated that in patients with suspected pulmonary embolism during COVID-19, CRT can be considered as a prognostic factor for right ventricular enlargement, especially as a negative predictor of right ventricular enlargement in the case of lower CTR values [29].

Summing up, it should be emphasized that despite the common belief that the CTR measurement value is low as a marker of LV hypertrophy, our research seems to fit in with the evidence provided by researchers in recent years regarding the predictive utility of the above radiological parameter. The strength of our study is also demonstrated that among the echocardiographically assessed types of left ventricular geometry, the radiological cardiothoracic ratio can be considered with high predictive accuracy as a marker of concentric hypertrophy and also as a marker of eccentric left ventricular hypertrophy. However, great caution should be exercised when trying to predict concentric remodeling of the left ventricular based on the radiological cardiothoracic ratio.

## 5. Conclusions

1. Radiological cardiothoracic ratio may be a moderate marker of left ventricular hypertrophy assessed according to standard echocardiographic criteria, provided that its cut-off point is standardized in each population of subjects.
2. Among the echocardiographically assessed types of left ventricular geometry, the radiological cardiothoracic ratio can be considered with high predictive accuracy as a marker of concentric hypertrophy and also as a marker of eccentric hypertrophy. Caution should be exercised in predicting concentric remodeling based on the radiographic cardiothoracic ratio.

## Figures and Tables

**Table 1:** Clinical characteristics of the study group.

	Mean	Standard deviation	Minimum value	Maximum value
Age (years)	49.52	9.64	30.00	65.00
Height (m)	1.67	0.09	1.49	1.89
Body mass (kg)	73.12	9.88	52.89	95.65
BMI (kg/m^2^)	26.30	2.78	20.56	32.31
BSA (m^2^)	1.82	0.16	1.49	2.15
Systolic BP (mmHg)	137.13	18.47	96.44	177.03
Diastolic BP (mmHg)	87.06	9.06	68.15	111.35
Total cholesterol (mg/dl)	199.90	33.65	111.01	308.39
HDL cholesterol (mg/dl)	53.15	6.62	29.02	86.40
LDL cholesterol (mg/dl)	104.70	15.16	23.19	209.75
Triglycerides (mg/dl)	125.26	36.76	52.83	315.58
Glucose (mg/dl)	121.71	42.83	75.00	312.00

	*Number*		*Percent*	

Men	38		39.6	
Women	58		60.4	
Normal body mass	27		28.1	
Overweight	61		63.5	
Obesity	8		8.3	
Arterial hypertension	86		89.6	
Dyslipidemia	59		61.5	
Type 2 diabetes	32		33.3	
Coronary artery disease	10		10.4	
Stroke	5		5.2	

BMI: body mass index; BP: blood pressure; BSA: body surface area; HDL: high-density lipoprotein; LDL: low-density lipoprotein.

**Table 2:** The results of imaging studies in the study group.

	Mean	Standard deviation	Minimum value	Maximum value
Echocardiography				
LVEDd (mm)	46.94	6.07	36.03	80.01
LVESd (mm)	35.88	6.54	22.44	53.77
IVSDd (mm)	11.91	1.02	9.47	14.21
PWDd (mm)	10.13	1.09	7.34	12.02
LVM (g)	193.44	47.31	97.17	484.71
LVMI (g/m^2^)	107.80	31.05	52.99	311.15
RWT	0.48	0.07	0.28	0.64

Chest radiograph in PA projection				

C width (mm)	167.24	19.98	131.00	223.00
T width (mm)	335.59	32.55	259.00	392.00
CTR	0.51	0.04	0.43	0.63

	*Number*		*Percent*	
Echocardiography				

NG	15		15.6	
LVH	81		84.4	
CR	47		49.0	
CH	15		15.6	
EH	19		19.8	

Chest radiograph in PA projection				
Heart silhouette enlargement (CTR > 0.50)	29		30.2	

C width: transverse dimension of the heart's silhouette; CH: concentric hypertrophy of the left ventricular; CR: concentric remodeling of the left ventricular; CTR: cardiothoracic ratio, EH: eccentric hypertrophy of the left ventricular; IVSDd: interventricular septum diastolic diameter; LVEDd: left ventricular end-diastolic diameter; LVESd: left ventricular end-systolic diameter; LVH: left ventricular hypertrophy; LVM: left ventricular mass; LVMI: left ventricular mass index; NG: normal geometry of the left ventricular; PWDd: posterior wall diastolic diameter; RWT: relative wall thickness; T width: transverse dimension of the chest.

**Table 3:** Geometry of the left ventricular in the studied subgroups differing in cardiothoracic ratio.

	Enlarged heart silhouette (CTR > 0.50)	Nonenlarged heart silhouette (CTR ≤ 0.50)	*p*
NG^a^	0.0 (0)	22.4 (15)	<0.05
LVH^a^	100.0 (29)	77.6 (52)	<0.05
CR^a^	37.9 (11)	53.7 (36)	ns
CH^a^	34.5 (10)	7.5 (5)	<0.05
EH^a^	27.6 (8)	16.4 (11)	ns

^a^Qualitative variable expressed as a percentage (number); CH: concentric hypertrophy of the left ventricular; CR: concentric remodeling of the left ventricular; CTR: cardiothoracic ratio; EH: eccentric hypertrophy of the left ventricular; LVH: left ventricular hypertrophy; NG: normal geometry of the left ventricular.

**Table 4:** Cardiothoracic ratio in the studied subgroups differing in the geometry of the left ventricular.

		CTR^b^	Heart silhouette enlargement (CTR >0.50)^a^
Subgroups differing in left ventricular hypertrophy	NG	0.46 ± 0.02	0.0 (0)
	LVH	0.52 ± 0.04	35.8 (29)
	*p*	<0.05	<0.05

Subgroups differing in the type of left ventricular geometry	NG	0.46 ± 0.02	0.0 (0)
	CR	0.49 ± 0.02	23.4 (11)
	CH	0.53 ± 0.04	66.7 (10)
	EH	0.52 ± 0.05	42.1 (8)
	*p*	NG vs. CR, CH, EH: *p* < 0.05	NG vs. CR, CH, EH: *p* < 0.05
		CR vs. CH, EH: *p* < 0.05	CR vs. CH: *p* < 0.05

^a^Qualitative variable expressed as a percentage (number); ^b^quantitative variable expressed as mean ± standard deviation; CH: concentric hypertrophy of the left ventricular; CR: concentric remodeling of the left ventricular; CTR: cardiothoracic ratio; EH: eccentric hypertrophy of the left ventricular; LVH: left ventricular hypertrophy; NG: normal geometry of the left ventricular.

**Table 5:** Correlation of the cardiothoracic ratio in the chest radiograph and the size of the left ventricular in the echocardiography.

	CTR	
	*r*	*p*
LVEDd (mm)	0.38	<0.05
LVESd (mm)	−0.12	ns
IVSDd (mm)	0.13	ns
PWDd (mm)	0.07	ns
LVM (g)	0.42	<0.05
LVMI (g/m^2^)	0.50	<0.50
RWT	−0.19	ns

CTR: cardiothoracic ratio; IVSDd: interventricular septum diastolic diameter; LVEDd: left ventricular end-diastolic diameter; LVESd: left ventricular end-systolic diameter; LVM: left ventricular mass; LVMI: left ventricular mass index; PWDd: posterior wall diastolic diameter; RWT: relative wall thickness.

**Table 6:** Sensitivity, specificity, and accuracy of the radiographic cardiothoracic ratio as a predictor of left ventricular geometry.

		Sensitivity	Specificity	Accuracy
Prediction of LVH	CTR > 0.50	1.000	0.358	0.458
	CTR > 0.49^*∗*^	0.933	0.827	0.844

Prediction of CR	CTR > 0.50	0.633	0.213	0.427
	CTR > 0.49^*∗*^	0.367	0.787	0.573

Prediction of CH	CTR > 0.50	0.778	0.667	0.760
	CTR > 0.52^*∗*^	0.840	0.600	0.802

Prediction of EH	CTR > 0.50	0.740	0.421	0.677
	CTR > 0.52^*∗*^	0.805	0.368	0.719

^*∗*^Optimal cut-off point according to the ROC curve; CH: concentric hypertrophy of the left ventricular; CR: concentric remodeling of the left ventricular; CTR: cardiothoracic ratio; EH: eccentric hypertrophy of the left ventricular; LVH: left ventricular hypertrophy.

## Data Availability

Study data can be made available upon documented request.
